# REAL-WORLD IMPLEMENTATION OF DEMENTIA CAREGIVING PROGRAMS: ALZHEIMER’S DISEASE PROGRAM INITIATIVE RESULTS

**DOI:** 10.1093/geroni/igae098.1685

**Published:** 2024-12-31

**Authors:** Heather Menne, Erin Long

**Affiliations:** Chair; Discussant

## Abstract

Since 1992 grants from the Administration on Aging have supported community services for people living with dementia and their caregivers. Beginning in 2014, grantees began addressing specific gaps in community-based care and services. Papers presented in this symposium highlight examples of each gap area. First, Yoon Chung Kim addresses the gap area of serving people who live alone with dementia and the successes and challenges faced in serving this population. The next two papers present examples of improving care and services for individuals aging with intellectual and developmental disabilities (IDD) with dementia or those at high risk of developing dementia. Dr. Kelli Barton discusses outcomes and lessons learned from the implementation of two dementia programs, Reducing Disability in Alzheimer’s Disease (RDAD) and Care Ecosystem, with the IDD population and their care partners. Dr. Faika Zanjani highlights the progress of the ADRD risk reduction health coaching delivered to older adults living with ADRD risk factors (e.g., diabetes, hypertension). Another gap area addresses the delivery of behavior symptom management training and consultation for family caregivers. Dr. Tamara Herrick discusses the impact and sustainability of the implementation of the Resources for Enhancing Alzheimer’s Caregiver Health (REACH) Community program within primary care and geriatrics settings. Dr. Christy Nishita presents the elements and successes of Hawaii’s memory care navigator model that educates, supports, and connects local families to the resources that they need. Erin Long, the Team Lead for Alzheimer’s and Dementia Programs at the Administration on Aging, Administration for Community Living offers discussion comments.

